# Does offering an incentive payment improve recruitment to clinical trials and increase the proportion of socially deprived and elderly participants?

**DOI:** 10.1186/s13063-015-0582-8

**Published:** 2015-03-07

**Authors:** Claudine G Jennings, Thomas M MacDonald, Li Wei, Morris J Brown, Lewis McConnachie, Isla S Mackenzie

**Affiliations:** Medicines Monitoring Unit (MEMO), University of Dundee, Ninewells Hospital, Dundee, DD1 9SY UK; School of Pharmacy, University College London, London, WC1E 6BT UK; University of Cambridge, Addenbrookes Centre for Clinical Investigation (ACCI), Addenbrookes Hospital, Cambridge, CB2 2QQ UK

**Keywords:** Clinical trial recruitment, Incentive payment, Patient demographics

## Abstract

**Background:**

Patient recruitment into clinical trials is a major challenge, and the elderly, socially deprived and those with multiple comorbidities are often underrepresented. The idea of paying patients an incentive to participate in research is controversial, and evidence is needed to evaluate this as a recruitment strategy.

**Method:**

In this study, we sought to assess the impact on clinical trial recruitment of a £100 incentive payment and whether the offer of this payment attracted more elderly and socially deprived patients. A total of 1,015 potential patients for five clinical trials (SCOT, FAST and PATHWAY 1, 2 and 3) were randomised to receive either a standard trial invitation letter or a trial invitation letter containing an incentive offer of £100. To receive payment, patients had to attend a screening visit and consent to be screened (that is, sign a consent form). To maintain equality, eventually all patients who signed a consent form were paid £100.

**Results:**

The £100 incentive offer increased positive response to the first invitation letter from 24.7% to 31.6%, an increase of 6.9% (*P* < 0.05). The incentive offer increased the number of patients signing a consent form by 5.1% (*P* < 0.05). The mean age of patients who responded positively to the invitation letter was 66.5 ± 8.7 years, whereas those who responded negatively were significantly older, with a mean age of 68.9 ± 9.0 years. The incentive offer did not influence the age of patients responding. The incentive offer did not improve response in the most socially deprived areas, and the response from patients in these areas was significantly lower overall.

**Conclusion:**

A £100 incentive payment offer led to small but significant improvements in both patient response to a clinical trial invitation letter and in the number of patients who consented to be screened. The incentive payment did not attract elderly or more socially deprived patients.

**Trial registrations:**

Standard care versus Celecoxib Outcome Trial (SCOT) (ClinicalTrials.gov identifier: NCT00447759).

Febuxostat versus Allopurinol Streamlined Trial (FAST) (EudraCT number: 2011-001883-23).

Prevention and Treatment of Hypertension with Algorithm Guided Therapy (British Heart Foundation funded trials) (PATHWAY) 1: Monotherapy versus dual therapy for initiating treatment (EudraCT number: 2008-007749-29).

PATHWAY 2: Optimal treatment of drug-resistant hypertension (EudraCT number: 2008-007149-30).

PATHWAY 3: Comparison of single and combination diuretics in low-renin hypertension (EudraCT number: 2009-010068-41).

**Electronic supplementary material:**

The online version of this article (doi:10.1186/s13063-015-0582-8) contains supplementary material, which is available to authorized users.

## Background

Efficient recruitment of patients into clinical trials is a major challenge in medical research. Recruited patients are often those interested in their health. The elderly, socially deprived and those with significant comorbidities are generally underrepresented. This raises concerns that the results of clinical trials may not be generalisable to all groups in the wider population and questions about the validity of using outcomes from these trials when making clinical decisions affecting the broader population [1,2].

Various methods have been used to improve recruitment into clinical trials. Recruitment strategies depend largely on the type of trial and the patient population required. Recruitment into studies with stringent inclusion and exclusion criteria can be particularly challenging. Traditional recruitment methods, including use of primary and secondary care practitioners, remain important; however, Investigators have also used public awareness campaigns and advertising through various media outlets to promote the benefits to society of participating in research and to attract a wider variety of patients [3-5].

The concept of using financial incentives to recruit patients into clinical trials is controversial, and attitudes toward them vary between countries and cultures. In the United Kingdom, healthy volunteers participating in research may be paid for their services [6]. For patients participating in clinical trials, there is agreement that they should be reimbursed for reasonable expenses; however, there is a clear distinction between appropriate reimbursement and additional payment as a financial incentive to participate. The situation is different in the United States, which has a largely insurance-based health care system. There, financial incentives are frequently used. A 2002 report found that in 32 US health care organisations surveyed, 58% of patients were paid an incentive to participate in research [7]. Researchers in the United States found that moderate incentive payments were effective at improving recruitment and were not seen as undue or unjust inducements [8].

Before further debate on the ethics of offering incentive payments to patients to participate in research, we felt it important to assess the effectiveness of incentive payments as a recruitment strategy in Scotland, both in terms of absolute numbers recruited and in terms of widening the demographic profile of those screened.

## Methods

We received ethical approval from the Scotland Research Ethics Committee (REC 12/SS/0006) to assess whether the offer of a fixed payment of £100 would improve recruitment into five different clinical trials currently running in the United Kingdom. The trials studied were the Standard care versus Celecoxib Outcome Trial (SCOT) [9], the Febuxostat versus Allopurinol Streamlined Trial (FAST) and the three British Heart Foundation–funded Prevention and Treatment of Hypertension with Algorithm Guided Therapy (PATHWAY) studies (1, 2 and 3). All trial participants provided us their written, informed consent to participate. Further details of the trials are shown in Table 1.

**Table 1:** **Overview of the five clinical trials used in this study** ^**a**^

**Trial**	**Sponsor**	**Outline**
SCOT [10] (ClinicalTrials.gov identifier: NCT00447759)	University of Dundee	Trial comparing the cardiovascular safety of celecoxib with that of other traditional NSAIDs in patients older than 60 years of age who are taking long-term NSAIDs for arthritis
FAST (EudraCT number: 2011-001883-23)	University of Dundee	Trial comparing the cardiovascular safety of febuxostat versus allopurinol in patients over the age of 60 years with symptomatic hyperuricaemia
PATHWAY 1 (EudraCT number: 2008-007749-29)	University of Cambridge	Trial of newly diagnosed hypertension in patients aged 18 to 79 years comparing monotherapy with dual therapy as initial hypertension treatment
PATHWAY 2 (EudraCT number: 2008-007149-30)	University of Cambridge	Trial investigating treatment of resistant hypertension in patients aged 18 to 79 years with uncontrolled blood pressure on three anti-hypertensive agents
PATHWAY 3 (EudraCT number: 2009-010068-41)	University of Cambridge	Trial comparing single-agent and combination diuretic therapy for low-renin hypertension in patients aged 18 to 80 years with at least one component of the metabolic syndrome

^a^FAST, Febuxostat versus Allopurinol Streamlined Trial; NSAID, Non-steroidal anti-inflammatory drug; PATHWAY, Prevention and Treatment of Hypertension with Algorithm Guided Therapy, British Heart Foundation–funded trials; SCOT, Standard care versus Celecoxib Outcome Trial.

For each of the five trials, potentially suitable patients were identified through a search of their general practitioners’ (GPs’) practice databases and invited to a screening visit. The invitation letters contained either the offer of the £100 incentive payment if the patient consented to be screened or a standard invitation letter with no incentive offer. If there was no response to the first invitation letter, a further letter was sent offering the incentive.

Sample size calculations were based on previous recruitment rates calculated from available data and differed between the five trials. For the FAST and SCOT studies combined, to detect a 50% increase in response to invitation letters at 80% power required 225 letters to be sent for each group. For the three PATHWAY studies, to detect a 50% increase would require 424 letters in each group; therefore, for practicality, it was decided to calculate for a 100% increase in response at 80% power, which required 121 invitation letters to be sent for each group. Owing to the difficulties in determining what would be considered a worthwhile increase in recruitment balanced against the cost of the incentive payment, sample size calculations were pragmatic.

To receive the payment, the patient had to attend a screening visit and consent to be screened for the trial (that is, sign a consent form). To maintain equity, the £100 incentive was paid to all patients who signed a consent form for any of the studies (without regard to whether the incentive offer was in the invitation letter). Eligible patients who wished to receive the payment provided bank details to the finance department of the Medicines Monitoring Unit of the University of Dundee, and the £100 payment was transferred directly into the patient’s bank account after the signed consent form was received.

Patients were randomised centrally from the Medicines Monitoring Unit of the University of Dundee. A list of suitable patients from each GP practice was generated by the research nurses for each trial. Patients on this list were randomly assigned a 0 (no incentive offer) or a 1 (incentive offer) using a computer algorithm (rand.nextdouble() > 0.500). This was a simple fixed randomisation method rather than an adaptive method; therefore, no effort was made to balance the groups. The code used generated random figures from ≥0 to <1. Figures ≤0.5 were assigned a 0, and figures >0.5 were assigned a 1. This led to a small unforeseen bias towards generating a 0 (no incentive offer). This imbalance was not evident until after the trial had been completed. Research nurses and study personnel were not blinded to the randomisation. Allocation of the incentive offer was independent for each trial.

Basic demographic information was recorded for each patient who was sent an invitation letter, including age, sex and postcode. The Scottish Index of Multiple Deprivation (SIMD) 2012 based on postcode was used as a measure of socioeconomic status [11]. The SIMD combines 38 indicators across 7 domains, including income, employment, health, education, skills, housing, access and crime. Although SIMD is not a perfect indicator, it was considered a reasonable way of estimating socioeconomic status for our trial populations, given the data we had available.

**Figure 1:**
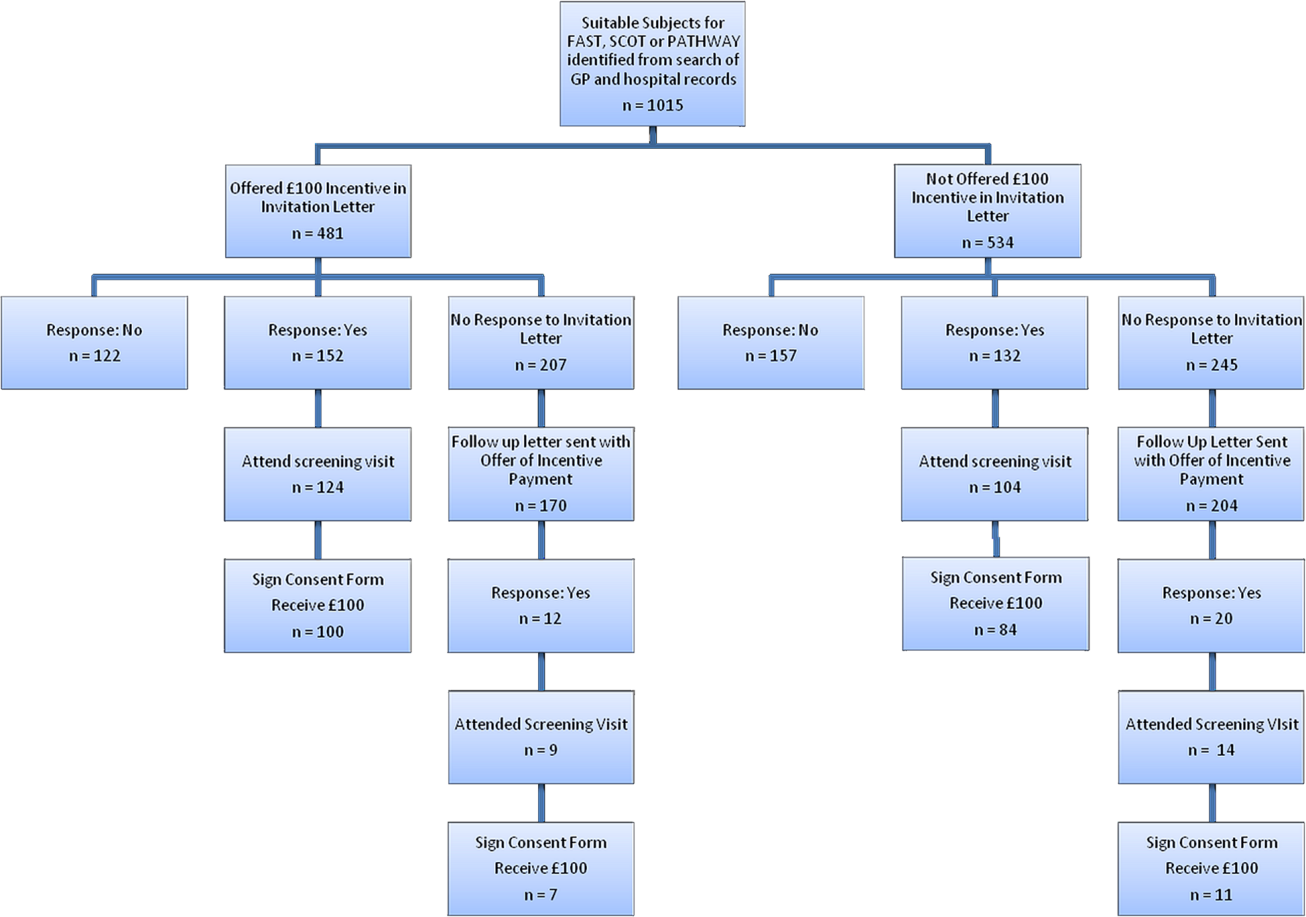
**Study schematic.** GP, General practitioner practice; FAST, Febuxostat versus Allopurinol Streamlined Trial; PATHWAY, Prevention and Treatment of Hypertension with Algorithm Guided Therapy, British Heart Foundation–funded trials; SCOT, Standard care versus Celecoxib Outcome Trial.

The primary outcome for this study was patient response to the first invitation letter, depending on whether the patient was offered the £100 incentive. Secondary outcomes included a comparison of the demographics of patients who responded positively between the incentive and non-incentive groups and the number of patients in each group who consented to be screened (and therefore received the incentive payment) and eventually were randomised into each study. The response to the follow-up letter was analysed separately. A study schematic with patient numbers is shown in Figure 1.

Data were summarised as mean and standard deviation for continuous variables and number of patients (percent) for categorical variables. The *χ*^2^ test and an independent *t*-test were performed to determine significant differences. Response rates to the first invitation letter were compared between the patients who were offered the incentive and patients who were not offered the incentive. A logistic regression model was employed to access factors affecting positive responses to the first invitation letter. Analysis was undertaken using SAS version 9.2 software (SAS Institute, Cary, NC, USA).

## Results

A total of 1,015 patients were sent a first invitation letter for one of the five clinical trials. This total comprised 332 (32.7%) patients for FAST, 181 (17.8%) patients for SCOT, 93 (9.2%) patients for PATHWAY 1, 210 (20.7%) patients for PATHWAY 2 and 199 (19.6%) patients for PATHWAY 3. A total of 481 (47.4%) patients were offered the incentive in the first invitation letter. The mean age of invited study subjects was 66.3 ± 9.9 years, and 58% of the patients were male. All patients were recruited in the East of Scotland (Dundee, Fife and Perth). There were no differences in age, sex, social deprivation, geographic location and invitation to different trials between the incentive and non-incentive groups (Table 2).

**Table 2:** **Patient characteristics** ^**a**^

	**Offered incentive (** ***N*** **= 481)**	**Not offered incentive (** ***N*** **= 534)**
Age (mean, SD)	66.2 (10.2)	66.3 (9.6)
Sex		
Male	281 (58.4)	308 (57.7)
Female	162 (33.7)	182 (34.1)
Unknown	38 (7.9)	44 (8.2)
SIMD deprivation category^b^		
1–3 (least deprived)	138 (28.7)	134 (25.1)
4–7	242 (50.3)	274 (51.4)
8–10 (most deprived)	101 (21.0)	125 (23.5)
Geographic areas^b^		
Angus and Dundee	217 (45.1)	215 (40.3)
Fife	200 (41.6)	234 (43.9)
Perth	64 (13.3)	84 (15.8)
Target patient group		
FAST	158 (32.9)	174 (32.6)
SCOT	84 (17.5)	97 (18.2)
PATHWAY 1	46 (9.6)	47 (8.8)
PATHWAY 2	101 (21.0)	109 (20.4)
PATHWAY 3	92 (19.1)	107 (20.0)

^a^FAST, Febuxostat versus Allopurinol Streamlined Trial; PATHWAY, Prevention and Treatment of Hypertension with Algorithm Guided Therapy, British Heart Foundation–funded trials; SCOT, Standard care versus Celecoxib Outcome Trial; SIMD, SD, Standard deviation; Scottish Index of Multiple Deprivation. Data are mean (SD) or number (%). There were no significant differences between groups ^b^Includes one patient with missing data.

### Primary outcome: response to first invitation letter

The response rates to the first invitation letter were 284 (28.0%) positive responses, 279 (27.5%) negative responses and 452 (44.5%) patients who did not respond at all. Table 3 shows the differences in positive responses between the incentive and non-incentive groups for each trial. Overall figures show there was a 6.9% increase in positive responses with the incentive offer (95% confidence interval (CI), 1.35 to 12.40; *P* = 0.04); however, there were marked differences in response rates for each of the five trials.

**Table 3:** **Invitation to screening visit and outcome of first invitation letter, by trial** ^**a**^

		**FAST (** ***N*** **= 332)**	**SCOT (** ***N*** **= 181)**	**PATHWAY 1 (** ***N*** **= 93)**	**PATHWAY 2 (** ***N*** **= 210)**	**PATHWAY 3 (** ***N*** **= 199)**	**Overall (** ***N*** **= 1,015)**
Offered incentive payment, n (%)		158 (47.6%)	84 (46.4%)	46 (49.5%)	101 (48.1%)	92 (46.2%)	481 (47.4%)
Not offered incentive payment, n (%)		174 (52.4%)	97 (53.6%)	47 (50.5%)	109 (51.9%)	107 (53.8%)	534 (52.6%)
Number of responses to first invitation letter	Positive						
	Incentive offer	68 (43.0%)	34 (40.5%)	5 (10.9%)	19 (18.8%)	26 (28.3%)	152 (31.6%)
	No incentive offer	54 (31.0%)	31 (32.0%)	7 (14.9%)	19 (17.4%)	21 (19.6%)	132 (24.7%)
	**% change with Incentive ((95% CI))**	**12.0%**	**8.5%**	−**4.0%**	**1.4%**	**8.7%**	6.9%* (1.35 to 12.40)
	Negative						
	Incentive offer	40 (25.3%)	15 (17.9%)	14 (30.4%)	30 (29.7%)	23 (25.0%)	122 (25.4%)
	No incentive offer	49 (28.2%)	16 (16.5%)	16 (34.0%)	37 (33.9%)	39 (36.4%)	157 (29.4%)
	**% change with incentive ((95% CI))**	−**2.9%**	−**1.4%**	−**3.6%**	−**4.2**	−**11.4%**	−**4.0% (−1.47 to 9.47)**
	No response						
	Incentive offer	50 (31.6%)	35 (41.7%)	27 (58.7%)	52 (51.5%)	43 (46.7%)	207 (43.0%)
	No incentive offer	71 (40.8%)	50 (51.5%)	24 (51.1%)	53 (48.6%)	47 (43.9%)	245 (45.8%)
	**% change with incentive ((95% CI))**	−**9.2%**	−**9.8%**	**7.6%**	**2.9%**	**2.8%**	−**2.8% (−3.27 to 8.92)**
Number of patients signing a consent form	Incentive offer	58 (36.7%)	26 (30.9%)	3 (6.5%)	4 (4.0%)	9 (9.8%)	100 (20.8%)
	No incentive offer	41 (23.6%)	24 (24.7%)	4 (8.5%)	9 (8.3%)	6 (5.6%)	84 (15.7%)
	**% change with incentive ((95% CI))**	**13.1%**	**6.2%**	−**2.0%**	−**4.3%**	**4.2%**	5.1%* (0.31 to 9.85)
Number of patients randomised into trial	Incentive offer	58 (36.7%)	26 (30.9%)	2 (4.3%)	3 (3.0%)	5 (5.4%)	94 (19.5%)
	No incentive offer	40 (23.0%)	24 (24.7%)	3 (6.4%)	6 (5.5%)	0	73 (13.7%)
	**% change with incentive ((95% CI))**	**13.7%**	**6.2%**	−**2.1%**	−**2.5%**	5.4%*	5.9%* (1.30 to 10.49)

^a^CI, Confidence interval; FAST, Febuxostat versus Allopurinol Streamlined Trial; PATHWAY, Prevention and Treatment of Hypertension with Algorithm Guided Therapy, British Heart Foundation–funded trials; SCOT, Standard care versus Celecoxib Outcome Trial. *P < 0.05.

A logistic regression model based on age, sex, incentive payment, trial, deprivation decile and geographic area showed that the incentive payment, age and invitation to the PATHWAY trials were significantly associated with response rates. Older patients and those invited to the PATHWAY trials were more likely to answer negatively to the first invitation letter (adjusted odds ratio, 0.95 (95% CI, 0.93 to 0.97) for age; 0.16 (0.07 to 0.39) for PATHWAY 1; 0.26 (0.14 to 0.50) for PATHWAY 2 and 0.29 (0.15 to 0.56) for PATHWAY 3).

### Secondary outcomes

#### Patient demographics

**Figure 2:**
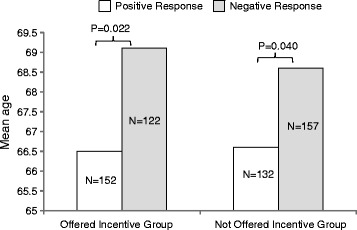
**Bar graph showing mean age (in years) of patients who responded positively versus negatively to the first invitation letter with versus without the incentive offer.**

The mean ages in years for the positive, negative and no-response groups for all trials are shown in Figure 2. Older patients were significantly more likely to respond negatively to the invitation letter, regardless of whether they were offered the incentive (*P* < 0.05). There are limitations in combining all trials for age, as the FAST and SCOT trials recruited only patients over the age of 60, whereas the PATHWAY trials were open to patients as young as 18 years of age. The oldest patients were in the FAST trial, with a mean age of 71.5 ± 7.6 years, and the youngest patients were in the PATHWAY 1 study, with a mean age of 57.9 ± 11.9 years. However, within each trial, older patients were numerically more likely to respond negatively to the first invitation letter; this reached statistical significance for the FAST, PATHWAY 1 and PATHWAY 3 studies. None of the trials showed that the offer of the incentive affected the age of the patients responding. Full results for the effect of age on responses by trial are shown in Additional file 1: Table S1.

Patients from more deprived areas (SIMD deciles 1–3) were less likely to respond positively to the invitation letter, with an overall positive response rate of 21.3% compared to an overall positive response of 31.4% from patients in the least deprived areas (SIMD deciles 8–10) (*P* = 0.032). There were no significant differences in positive response to the first invitation letter with or without the incentive offer in the most deprived areas or in the least deprived areas; however, patients in SIMD deciles 4–7 were significantly more likely to respond positively to the incentive offer (37.2% versus 24.0%) (*P* = 0.004). Full results are available in Additional file 2: Table S3.

#### Patients consented and randomised

In total, 284 patients responded positively to the first invitation letter, and 184 (64.8%) of these signed a consent form (making them eligible for the incentive payment). Of these 184 patients, 100 who signed a consent form were offered the incentive, which represents a 5.1% increase in consented patients who received the incentive offer (*P* = 0.037). Of the 184 consented patients, 167 were ultimately randomised into a trial. Table 3 shows the differences in consented and randomised patients by trial. The greatest (although not statistically significant) positive changes with the incentive offer were seen in the FAST trial.

### Response to non-responder letters

Non-responder letters were sent to 374 of the 452 non-responders to the first letter (full results are available in Additional file 3: Table S3). The non-responder letter contained the offer of the £100 incentive; therefore, all non-responders were offered the incentive. The non-responder letters generated a further 66 responses (17.4%), of which 32 (8.6%) were positive responses; among the latter group of 32 patients, 18 patients signed a consent form and 14 were randomised into a trial. The overall response rate (both positive and negative) for the first invitation letter and the follow-up letter for the 1,015 patients contacted in this trial was 62%.

### Overall outcomes for all patients

Final figures for both the first and the non-responder letters show that, in total, there were 316 patients who responded positively to the invitation letter (184 offered the incentive, 132 not offered the incentive). Of these 316 patients, 251 attended a screening visit. A total of 202 patients signed a consent form and were eligible for the £100. Ultimately, 181 patients were randomised into a study (104 for FAST, 58 for SCOT, 5 for PATHWAY 1, 9 for PATHWAY 2 and 5 for PATHWAY 3). Thus, after writing to 1,015 patients, 181 were ultimately randomised into a trial, giving an overall randomisation rate of 17.8%.

### Cost-effectiveness

The additional cost incurred in undertaking this incentive payment trial was £100 per patient who signed a consent form, as invitation letters, trial information and screening visits were unchanged from the usual recruitment process of each trial. With 202 patients consented into the trials, the total cost of the payments to patients in the trial was £19,900 (3 patients did not accept the incentive payment).

The cost to each trial of paying a £100 incentive is determined by the number of patients signing the consent form (as only these patients are paid). Some patients who responded positively to the invitation letter did not meet trial inclusion criteria, however; therefore, despite their wish to participate and claim the £100, they were ineligible. Table 4 shows the cost for each additional patient who responded positively to the invitation letter, as well as the cost for each consenting patient if the incentive was offered. Overall, the cost was £1.549 to get one additional patient to respond positively; however, significant differences between the trials were again evident.

**Table 4:** **Cost per additional responding or consented patient from first invitation letter by trial** ^**a**^

	**Increase in positive response with incentive**	**Cost per additional patient**	**Increase in consented patients with incentive**	**Cost per additional consented patient**
Overall	6.9%	£1,549	5.1%	£1,961
FAST	12.0%	£933	13.1%	£763
SCOT	8.5%	£1,276	6.2%	£1,613
PATHWAY 1	−4.0%	N/A	−2.0%	N/A
PATHWAY 2	1.4%	£7,243	−4.3%	N/A
PATHWAY 3	8.7%	£1,249	4.2%	£2,381

^a^FAST, Febuxostat versus Allopurinol Streamlined Trial; N/A, Not applicable; PATHWAY, Prevention and Treatment of Hypertension with Algorithm Guided Therapy, British Heart Foundation–funded trials; SCOT, Standard care versus Celecoxib Outcome Trial.

## Discussion

Over one-third of the patients invited to participate in a clinical trial did not respond at all to the invitation letter. This lack of response is commonplace when attempting to recruit participants for research and means effective clinical trial recruitment is difficult.

The offer of a £100 incentive payment did have some impact in improving patient response to the first invitation letter, and positive responses increased by 6.9% in the incentive group. The improvement in initial positive response did lead to a small (5.1%) increase in the number of patients signing a consent form. These increases were statistically significant when looking at only the response to the first invitation letter, but they became non-significant when overall figures for both the first and non-responder letters were used, owing to the poor response to the non-responder letters.

The five trials included in this study targeted different patient populations and required different levels of input from recruited patients; therefore, response rates varied significantly between the different trials. The recruitment for the SCOT and FAST trials was through GP’s writing to patients, and participants had to be older than 60 years of age and meet other entry criteria regarding their medication and medical history. SCOT and FAST were streamlined trials with the aim of replicating day-to-day clinical care; therefore, patients attended a screening visit and were then followed up remotely, so the burden on participants was relatively small. The PATHWAY studies recruited patients from primary care who were aged 18–80 years with either newly diagnosed or partially treated hypertension. All three PATHWAY studies involved multiple patient visits to the study centre as well as home blood pressure monitoring. The PATHWAY trials therefore often targeted a younger, working patient group and required a significantly greater investment in terms of time and effort from participants. Overall, initial positive response rates to SCOT and FAST invitations (both with and without the incentive) were 37% and 36%, respectively, compared to 19% for the PATHWAY trials combined. The greatest increase in patients actually randomised into a trial with the incentive offer was seen for the FAST trial, but this 13.7% increase was not statistically significant. Subsequent screening and randomisation rates were also significantly poorer for all of the PATHWAY studies, which was due to stringent study entry criteria for blood pressure as well as the greater perceived burden of multiple study visits for the participants. Recruitment into PATHWAY 1 and 2 appears to have been negatively affected by the incentive offer; however, the number of consented patients was too small to allow meaningful interpretation. Recruitment into PATHWAY 3 increased with the incentive offer, but this still represented only 5 patients randomised out of 199 patients contacted.

Cost-effectiveness was calculated as the cost of one additional responding or consented patient if all patients received £100. The FAST trial showed the greatest increase in consented patients with the incentive offer; however, even with a 13.1% increase, the cost per additional patient was still high at £763. It would be beyond the scope of most studies, particularly those lacking commercial funding, to view this as cost-effective.

The cost-effectiveness analysis is also perhaps unfair because the calculations were based only on the number of patients who received the payment. Patients who did not meet study inclusion criteria were never in a position to be able to receive the incentive payment, even though they had indicated a willingness to take part in a clinical trial. Therefore, cost-effectiveness figures could be marginally improved if investigators invited fewer unsuitable patients to screening visits.

The results of our present study indicate that the offer of a £100 financial incentive had only a limited impact on improving recruitment into the five clinical trials involved. This may initially appear surprising, as most people would acknowledge that we are all driven to some extent by the attainment of financial reward. However, in the health care setting, people are motivated to different degrees by personal gain and altruism. In 1970, Richard Titmuss claimed that paying people for blood donations actually made them less likely to donate, as the payment removed the altruistic incentive [12]. It was then suggested that increasing the payment would be effective; however, subsequent work has shown that payment can reduce intrinsic motivation and that people are often distrustful of payment offered for altruistic behaviour, including participation in clinical trials [13]. There is significant individual variation in how people respond to financial incentive and complex reasons underpinning these decisions. What may seem a worthwhile reward for some people may not attract others and may even actively discourage people who are uncomfortable with receiving financial reward for altruistic behaviour.

The incentive offer for this study was £100. This figure was chosen as a compromise between reasonable compensation for the degree of inconvenience caused against an effective incentive to participate. The level of the incentive offered must fulfil the primary objective of increasing recruitment without being seen as financial coercion. Studies done in the United States have shown that the level of payment does influence response. For example, in a study in which researchers looked at enrolling teenagers in a smoking cessation program, the results showed that any incentive was better than no incentive and that a $15 cash incentive improved responses compared to a $2 cash incentive and entry into a $200 prize drawing [3,10]. Incentive payments in clinical research are controversial, particularly regarding where to draw the line between financial incentive and financial coercion. This concern is especially relevant when trying to recruit socially deprived and vulnerable members of society [14].

There has also been debate over whether the size of the payment should reflect the risk or inconvenience of the trial and if a standard formula could be developed to determine the amount of the incentive [15,16]. The five trials in this study were all considered low-risk (all used medicines within their licensed indications), and therefore no assessment was made of the participants’ views of the risks involved and whether this influenced their decision to participate. Opinions are divided on this topic, with many people feeling that higher-risk trials should not offer incentive payments at all [17]. Researchers in the United States have looked at how patients respond to payment offers in terms of both the level of payment and the perceived risk associated with the trial. Halpern and colleagues conducted a study where 126 hypertensive patients were provided with information on a series of potential trials of a new anti-hypertensive medication. In a 3 × 3 design, the risk of adverse events and the payment offer ($100, $1,000 or $2,000) were varied. In 34% of patients, the level of payment significantly influenced their willingness to participate. Unsurprisingly, trials with a higher risk of side effects and lower payment offers decreased willingness to participate. They also found a non-significant trend towards wealthier people being more strongly influenced by payment, but they did not find that patients’ perceptions of the risks associated with the trials were altered by the level of the payment offered [8].

Our study shows that, though there may be some case to be made for incentive payments’ marginally improving recruitment into some trials, there was no evidence that the incentive payment broadened the demographics of those participating. The incentive payment offer had no impact on the age of those responding, and, for every trial within the study, there was a trend towards older patients responding negatively to the invitation letter and younger patients not responding at all (irrespective of the incentive offer). This may reflect younger patients having work and family commitments that prevent their participation and older patients having different priorities, such as increased frailty that prevents multiple study visits and presence of multiple comorbidities that make these patients less willing to spend additional time with health care professionals.

In terms of social deprivation, the initial positive response (with or without the incentive offer) from patients in the most deprived areas (SIMD deciles 1–3) was 21.3%, which was lower than the overall trial average of 28.0%. Positive responses from patients in the least deprived areas (SIMD deciles 8–10) were higher than the trial average at 30.7%. Patients in the middle deprivation deciles (SMID deciles 4–7) were the only group to show a significant increase in positive responses associated with the incentive payment. We can only speculate on the reasons for this. It might be that the most deprived in society are the least engaged with health care, whereas those who are better off would not be enticed by a relatively small payment but take a greater interest in their health.

There are limitations to this trial, including looking at patient recruitment from part of eastern Scotland only. Using five diverse clinical trials complicated interpretation of the results, as recruitment varied widely between trials. There are likely to have been different factors affecting recruitment within each trial, including how the trial was advertised and how patients were approached. It might have been interesting to look at different levels of payment; however, it would be expected that a lower figure would have made even less impact on patient recruitment and that a higher figure would mean the trial was not financially viable. Future work in this area could look at incentives that are not purely financial and are individualised to patients or trials.

## Conclusions

Both simple and complex messages emerge from this study. Put simply, paying patients £100 did entice more people to respond positively to an invitation letter and did result in slightly more randomised patients, particularly in the FAST trial; however, this effect was marginal. Response rates varied a great deal between the different trials, and, even where a significant improvement was observed, it would be a stretch to see this as a cost-effective recruitment method. The incentive payment did not attract the elderly or the more socially deprived. What motivates people to participate in clinical research remains elusive to the research community, and it would appear that £100 is not sufficient motivation for most.

## Additional files

Additional file 1: Table S1. Age of patients responding to first invitation letter by trial. Additional file 2: Table S2. Positive response to first invitation letter with versus without incentive offer by deprivation score. Additional file 3: Table S3. Response to non-responder letter (all patients are offered a £100 incentive in non-responder letter).

## Abbreviations

CI: Confidence interval
FAST: Febuxostat versus allopurinol streamlined trial
NSAID: Non-steroidal anti-inflammatory drug
PATHWAY: Prevention and treatment of hypertension with algorithm guided therapy, British heart Foundation–funded trials
SCOT: Standard care versus celecoxib outcome trial
SIMD: Scottish index of multiple deprivation
