# Supplementary material for: Does offering an incentive payment improve recruitment to clinical trials and increase the proportion of socially deprived and elderly participants?
Source: Trials. 2015 Mar 7;16:80. doi: 10.1186/s13063-015-0582-8 (PMC4364332; doi:10.1186/s13063-015-0582-8)
Supplement: Additional file 1: Table S1. — Age of patients responding to first invitation letter by trial. [file 13063_2015_582_MOESM1_ESM.doc]

Additional file 1: Table S1 Age of patients responding to first invitation letter by trial

| Trial | Offered Incentive | Response to First Letter | Number of Patients | Mean Age (years) | p value | |
| --- | --- | --- | --- | --- | --- | --- |
| FAST | Yes | Yes | 68 | 69.9 | 0.002 | 0.003 |
|  |  | No | 40 | 74.5 |  |  |
|  |  | No Response | 50 | 71.5 |  |  |
|  | No | Yes | 54 | 70.4 | 0.221 |  |
|  |  | No | 49 | 72.1 |  |  |
|  |  | No Response | 71 | 71.9 |  |  |
| SCOT | Yes | Yes | 34 | 66.3 | 0.990 | 0.81 |
|  |  | No | 15 | 66.3 |  |  |
|  |  | No Response | 35 | 68.4 |  |  |
|  | No | Yes | 31 | 66.4 | 0.697 |  |
|  |  | No | 16 | 67.0 |  |  |
|  |  | No Response | 50 | 66.0 |  |  |
| PATHWAY 1 | Yes | Yes | 5 | 53.0 | 0.027 | 0.017 |
|  |  | No | 14 | 66.6 |  |  |
|  |  | No Response | 27 | 55.7 |  |  |
|  | No | Yes | 7 | 58.6 | 0.283 |  |
|  |  | No | 16 | 63.5 |  |  |
|  |  | No Response | 24 | 52.5 |  |  |
| PATHWAY 2 | Yes | Yes | 19 | 65.0 | 0.504 | 0.056 |
|  |  | No | 30 | 67.1 |  |  |
|  |  | No Response | 52 | 62.9 |  |  |
|  | No | Yes | 19 | 64.0 | 0.036 |  |
|  |  | No | 37 | 68.9 |  |  |
|  |  | No Response | 53 | 63.3 |  |  |
| PATHWAY 3 | Yes | Yes | 26 | 61.5 | 0.150 | 0.016 |
|  |  | No | 23 | 65.9 |  |  |
|  |  | No Response | 43 | 59.8 |  |  |
|  | No | Yes | 21 | 62.2 | 0.078 |  |
|  |  | No | 39 | 66.8 |  |  |
|  |  | No Response | 47 | 60.0 |  |  |
