# Supplementary material for: Does offering an incentive payment improve recruitment to clinical trials and increase the proportion of socially deprived and elderly participants?
Source: Trials. 2015 Mar 7;16:80. doi: 10.1186/s13063-015-0582-8 (PMC4364332; doi:10.1186/s13063-015-0582-8)
Supplement: Additional file 2: Table S2. — Positive response to first invitation letter with versus without incentive offer by deprivation score. [file 13063_2015_582_MOESM2_ESM.doc]

Additional file 2: Table S2 Positive response to first invitation letter with and without incentive offer by deprivation score

|  | Deprivation Decile | FAST  (n=331) | SCOT  (n=181) | PATHWAY 1 (n=93) | PATHWAY 2 (n=210) | PATHWAY 3 (n=199) | OVERALL |
| --- | --- | --- | --- | --- | --- | --- | --- |
| Number of Patients  [=offered incentive] | 1-3  4-7  8-10 | 48 [27]  220 [105]  63 [26] | 17 [6]  134 [65]  30[13] | 48 [25]  27 [14]  18 [7] | 88 [44]  68 [32]  54 [25] | 71 [36]  67 [26]  61 [30] | 272 [138]  516 [242]  226 [101] |
| Positive Response with incentive | 1-3  4-7  8-10 | 11 (40.7%)  49 (46.7%)  8 (30.8%) | 1 (16.7%)  28 (43.1%)  5 (38.5%) | 4 (16.0%)  1 (7.1%)  0 (0%) | 8 (18.2%)  2 (6.3%)  9 (36.0%) | 7 (19.4%)  10 (38.5%)  9 (30.0%) | 31 (22.5%  90 (37.2%)  31 (30.7%) |
| Positive Response without incentive | 1-3  4-7  8-10 | 8 (38.1%)  31 (27.0%)  15 (40.5%) | 2 (18.2%)  20 (29.0%)  9 (52.9%) | 2 (8.7%)  4 (30.8%)  1 (9.1%) | 7 (15.9%)  4 (11.1%)  8 (27.6%) | 8 (22.9%)  6 (14.6%)  7 (22.6%) | 27 (20.1%)  65 (23.7%)  40 (32.0%) |
| **Change in positive response with incentive** | 1-3  4-7  8-10 | +2.6%  +19.7%  -9.7% | -1.5%  +14.1%  -14.4% | -7.3%  -23.7%  -9.1% | +2.3%  -4.8%  +8.4% | -3.5%  +23.9%  +7.4% | **+2.4%**  **+13.5%****  **-1.3%** |

Data for 1014 patients (1 missing data from FAST trial)

Deprivation Decile based on Scottish Index of Multiple Deprivation data 2012 (Decile 1 = Most deprived, Decile 10 = Least Deprived)

** significant <0.005
