# Supplementary material for: Does offering an incentive payment improve recruitment to clinical trials and increase the proportion of socially deprived and elderly participants?
Source: Trials. 2015 Mar 7;16:80. doi: 10.1186/s13063-015-0582-8 (PMC4364332; doi:10.1186/s13063-015-0582-8)
Supplement: Additional file 3: Table S3. — Response to non-responder letter (all patients are offered a £100 incentive in non-responder letter). [file 13063_2015_582_MOESM3_ESM.doc]

Additional file 3: Table S3 Response to non-responder letter (all patients are offered £100 incentive in non-responder letter)

|  | | FAST | SCOT | PATHWAY 1 | PATHWAY 2 | PATHWAY 3 | OVERALL |
| --- | --- | --- | --- | --- | --- | --- | --- |
| Non-Responders to First Letter, n | | 121 | 85 | 51 | 105 | 90 | 452 |
| Non-Responder Letters Sent, n† | | 112 | 57 | 45 | 90 | 70 | 374 |
| Response to Non-Responder Letter | **POSITIVE** | 11 (9.8%) | 9 (15.8%) | 0 | 5 (5.6%) | 7 (10.0%) | 32 (8.6%) |
|  | **NEGATIVE** | 21 (18.8%) | 9 (15.8%) | 0 | 1 (1.1%) | 3 (4.3%) | 34 (9.1%) |
|  | **NO RESPONSE** | 80 (71.4%) | 39 (68.4%) | 45 (100%) | 84 (93.3%) | 60 (85.7%) | 308 (82.3%) |
| Consent Form Signed | | 6 | 8 | 0 | 1 | 3 | 18 |
| Randomise into Trial | | 6 | 8 | 0 | 0 | 0 | 14 |

† 78 non-responders were not sent a non-responder letter
